# 3D-printed custom implant for the management of “locked” posterior dislocation of the shoulder joint with reverse Hill-Sachs lesion: a case report

**DOI:** 10.3389/fbioe.2023.1259255

**Published:** 2023-10-09

**Authors:** Yongrong Hu, Kunhai Yang, Hao Liu, Liping Wang, Song Wang, Xiang Zhang, Bo Qu, Hongsheng Yang

**Affiliations:** Department of Orthopedics, Clinical Medical College and The First Affiliated Hospital of Chengdu Medical College, Chengdu, Sichuan Province, China

**Keywords:** reverse Hill-Sachs lesion, posterior dislocation, electric shock injuries, 3D printing, shoulder joint

## Abstract

**Introduction:** Irregular bone defects of the humerus are common in clinical practice, but there are fewer reported cases of irregular humeral defects accompanied by shoulder joint “locking” dislocation and reverse Hill-Sachs injury caused by an electric shock. The choice of treatment for such cases is closely related to the extent of shoulder joint function recovery. This is a case report of a 60-year-old male patient who suffered from a shoulder joint “locking” dislocation with accompanying reverse Hill-Sachs injury due to muscle contraction after being electrically shocked at work. The patient was treated with a 3D-printed custom humeral head prosthesis for the treatment of the shoulder joint “locking” dislocation and reverse Hill-Sachs injury.

**Case presentation:** A 60-year-old male patient, working as a construction worker, presented to our emergency department with right shoulder pain and restricted movement for more than 30 min after an electric shock. Right humeral CT revealed a comminuted fracture of the right humeral head. D-dimer levels were significantly elevated at 3239.00 ng/mL, and oxygen partial pressure was slightly decreased at 68 mmHg. Treatment included emergency wound debridement and dressing for the electrical injury, cardioprotective measures, anticoagulation, and symptomatic management. After stabilizing the patient’s condition, the patient underwent 3D-printed custom prosthesis-assisted partial replacement of the right humeral head and rotator cuff repair in the orthopedic department. Postoperatively, the patient’s right shoulder joint wound healed well, and mobility was restored.

**Conclusion:** This case report demonstrates that the use of a 3D-printed custom prosthesis for the treatment of irregular humeral bone defects caused by specific injury mechanisms, especially cases involving shoulder joint “locking” dislocation and reverse Hill-Sachs injury, can achieve precise bone defect repair, minimize surgical trauma, and provide superior outcomes in terms of postoperative functional rehabilitation.

## Introduction

Electrical injuries pose significant risks to the human body and can result in multisystem damage, including injuries to the musculoskeletal, respiratory, cardiovascular, and central nervous systems (Zhang et al., 2017). The injuries caused by electric shocks are primarily characterized by burns but can also lead to secondary damage such as fractures and dislocations. These secondary injuries often occur due to loss of consciousness and subsequent falls or as a result of involuntary muscle contractions (Kokkalis et al., 2017).

The shoulder joint, being the most mobile and unstable joint in the human body, is particularly susceptible to injuries following an electric shock. The surrounding muscles of the shoulder joint forcefully contract, pulling on the bones and causing posterior dislocation of the shoulder joint along with an anterior fracture of the humeral head. This condition, known as “locking” dislocation of the shoulder joint with associated reverse Hill-Sachs injury, presents challenges in terms of diagnosis and treatment, with literature reporting a misdiagnosis and missed diagnosis rate of up to 60% (Stone et al., 2014). This injury has been labeled a “treatment trap” due to its complex nature.

Irregular large bone defects of the humeral head present a significant challenge for orthopedic surgeons. Traditional methods of fracture fixation and shoulder joint replacement have shown limitations in long-term outcomes for irregular large bone defects accompanied by “locking” dislocation of the shoulder joint and reverse Hill-Sachs injury. However, with the rapid development of digital technology, 3D printing technology, also known as additive manufacturing, has emerged as a promising approach for surgical planning and preoperative simulations in cases of humeral head injuries.

In this case report, we present the case of a 60-year-old male patient admitted to our hospital following an electrical injury. The patient underwent treatment for a fractured right humeral head using a 3D-printed custom prosthesis. Given the unique mechanism of injury and the complexity of the case, this report aims to emphasize the necessity and importance of utilizing 3D-printed custom prostheses in the treatment of “locking” dislocation of the shoulder joint with associated reverse Hill-Sachs injury and irregular large bone defects of the humeral head. The report provides a detailed description of the patient’s diagnostic and treatment process.

## Case presentation

A 60-year-old middle-aged laborer had been engaged in construction activities at the worksite, required to use iron tools to mix concrete within a cement-filled barrel. Unfortunately, the construction site’s electrical wires accidentally came into contact with the cement-filled iron barrel. The electrical current from the wires was conducted through the iron barrel into his body, resulting in his electrocution (approximately 380V) and subsequent collapse. He immediately experienced chest tightness, blurred vision, and excruciating pain that immobilized his right shoulder. Promptly, his colleagues dialed emergency services (120) and swiftly transported him to the Emergency Department of the First Affiliated Hospital of Chengdu Medical College. Upon arrival, a burn care physician conducted a physical examination, noting significant tenderness and limited mobility in the right shoulder joint. However, the Dugas sign yielded a negative result (−). Furthermore, the patient had multiple electrical burn wounds on his hands and feet, characterized by volcano-shaped lesions with dry, black charred tissue inside. After confirming the patient’s stable vital signs, the wounds underwent debridement and dressing. The patient received treatment at the Burn Unit. Following treatment there, the patient was further transferred to the Orthopedics Department for further evaluation.

**FIGURE 1:**
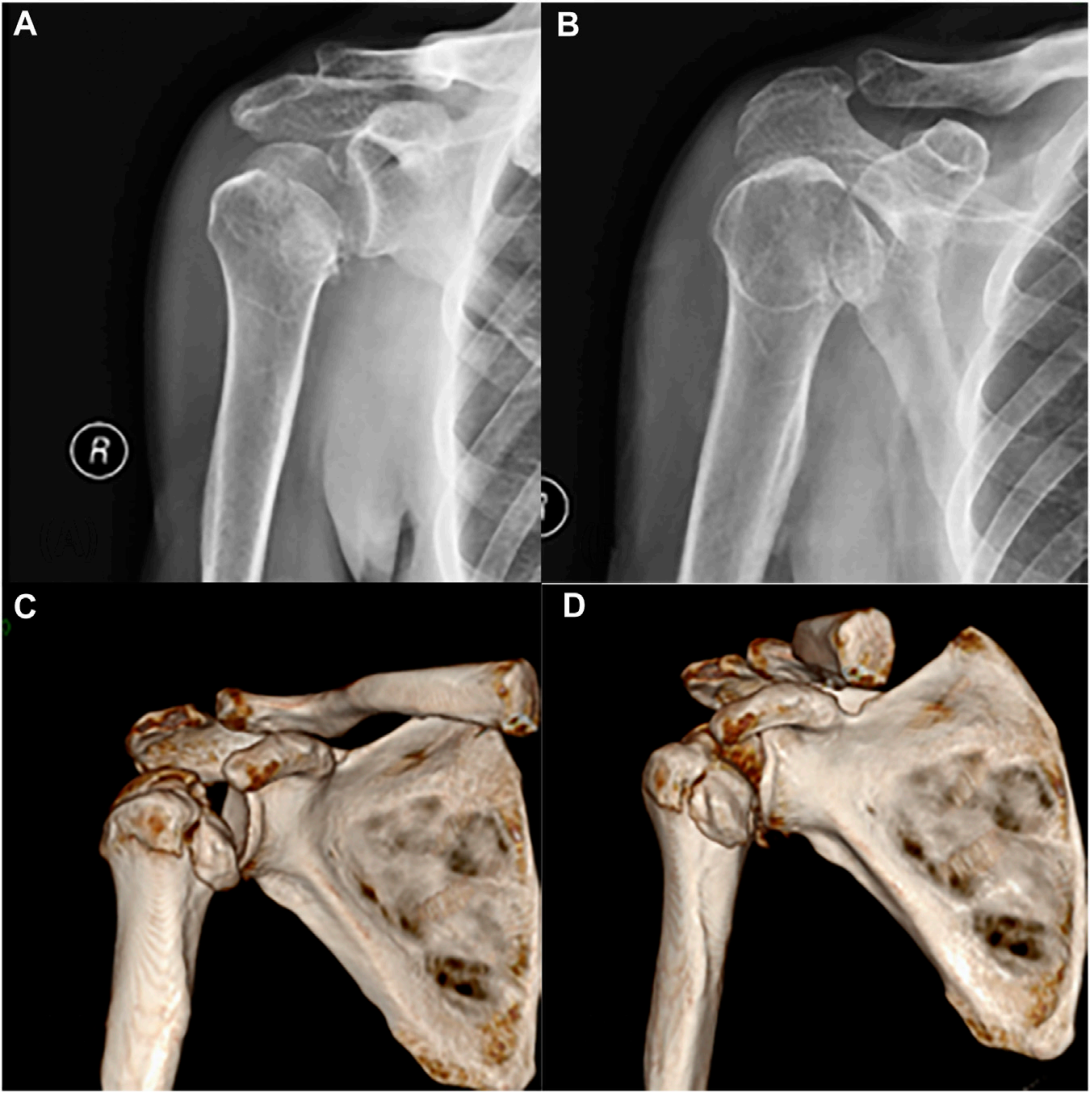
**(A, B)** Preoperative X-ray and **(C, D)** CT bone three-dimensional reconstruction.

Radiographic and three-dimensional CT scans were performed to assess the condition of the patient’s right shoulder joint (Figure 1). The imaging revealed a comminuted fracture of the right humeral head with fragmented, collapsed fragments and surrounding soft tissue swelling, along with a minor amount of air. Preoperative examination demonstrated significant impairment in the patient’s range of motion in the right shoulder joint. General information and indicators of abnormal laboratory tests upon admission see in Table 1.

**TABLE 1:** General information and indicators of abnormal laboratory tests upon admission.

Category	Result	Reference value	Counting unit
Gender	Male	—	—
Age	60	—	—
Past Medical History	none	—	—
Family History	none	—	—
Personal History	none	—	—
Blood Routine			
WBC	14.26	4–10	10^9/L
NE%	80.2	45–77	%
LY%	10.1	20–40	%
MO%	9.3	3–8	%
NE#	11.43	2–7.7	10^9/L
MO#	1.33	0.12–0.8	10^9/L
Liver Function, Renal Function and Cardiac Enzymes			
TP	55.6	65–85	g/L
ALB	35.2	40–55	g/L
CK	392	<200	U/L
Urine Analysis			
PRO	+-	-	—
PH	8.0	5.0–7.5	—
BLD	3+	-	—

Based on the patient’s medical history and auxiliary examinations, the orthopedic physician established the following diagnoses: 1) Comminuted fracture with a substantial bone defect in the right humeral head, 2) “Locking” dislocation of the right shoulder joint accompanied by a reverse Hill-Sachs injury, 3) Proximal fracture of the right humerus, and 4) Electrical injury with necrotic electrical burns on both hands and feet. After careful deliberation, the medical team decided to proceed with a 3D-printed custom prosthesis-assisted surgery, which involved partial replacement of the right humeral head and exploration and repair of the rotator cuff, once the patient’s condition stabilized. A 3D-printed model was used to simulate the surgical procedure, enabling the formulation of an effective plan that would address the remaining fracture fragments specific to the patient’s case. During the design phase of the 3D-printed prosthesis, various factors were taken into account, including prosthesis compatibility, the arrangement of rotator cuff suture holes, and the integration of the prosthesis and bone contact surface to promote bone integration.

The 3D-printed prosthetic limb was fabricated using Ti-6Al-4V material, renowned for its outstanding biocompatibility and bone integration properties (Li et al., 2020). The prosthetic limb was designed utilizing Mimics software (version 20.0; Materialise, Belgium) and manufactured through 3D printing technology by Chunli Limited Company (Beijing, People’s Republic of China). Before proceeding with final production, the prosthetic limb model underwent printing and testing procedures to validate our design. The entire process, commencing from the collection of patient data to the production of the prosthetic limb, typically spanned approximately 3 weeks.

**FIGURE 2:**
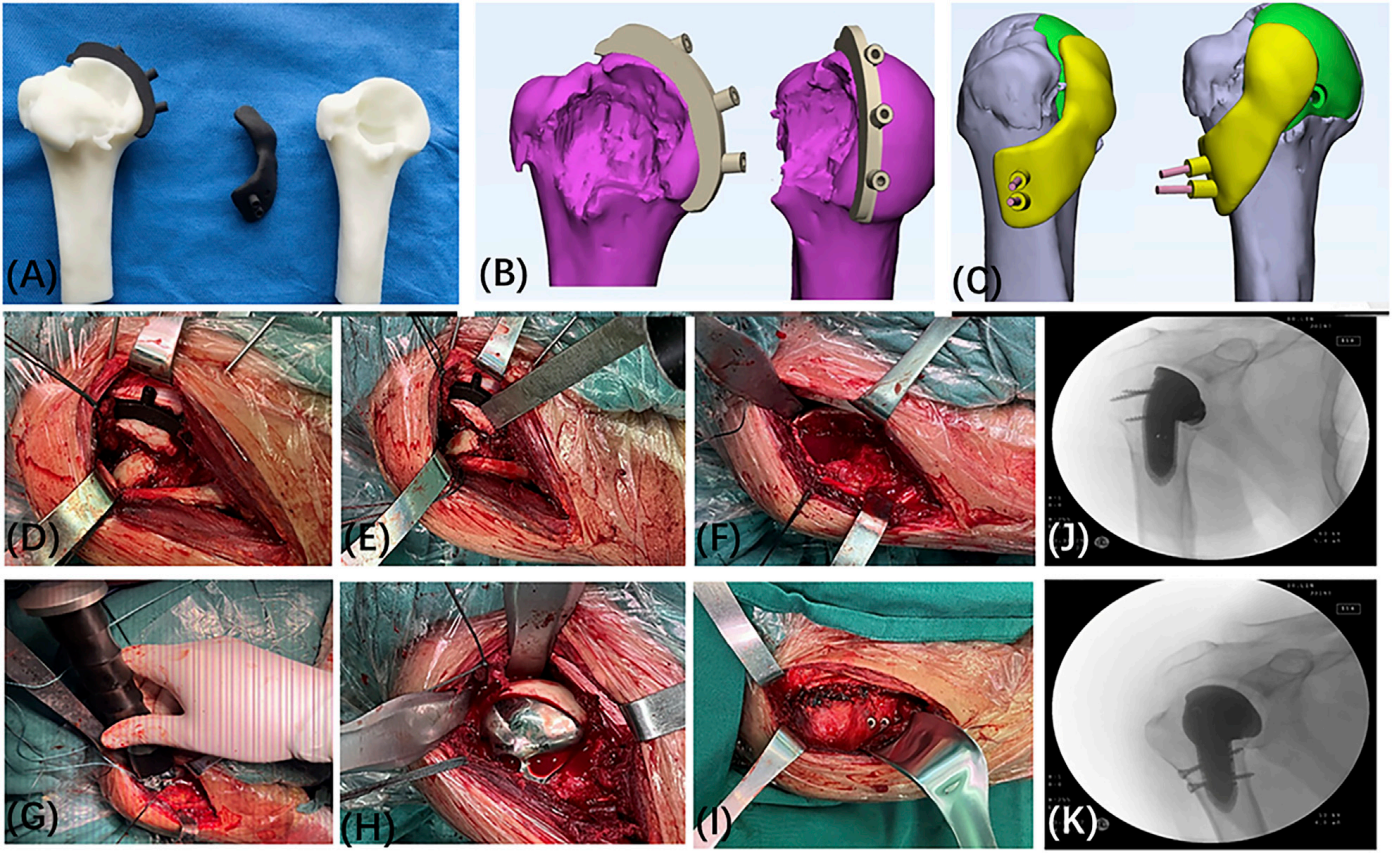
Custom 3D Printed Prosthesis Design and Surgical Procedure. **(A–C)** Preoperative Simulated Guide Plate Osteotomy and Fixation. **(D–F)** During the surgery, a significant bone defect was observed in the right humeral head. **(G–I)** Intraoperatively, the site of the humeral head injury and the position of the 3D-printed implant were confirmed. Repairs were performed on the rotator cuff and the insertion point of the infraspinatus muscle on the scapula. **(J, K)** Intraoperatively, fluoroscopy shows that the implant is well-placed.

The surgical procedure (Figure 2) was carried out as follows: The surgical site was accessed through the deltopectoral groove of the pectoralis major muscle. During the surgery, the greater and lesser tuberosities of the right humerus were exposed, revealing a severe fracture with small and fragmented bone pieces that could not be adequately stabilized using screws. The right humeral head was dislocated and trapped, necessitating the use of a bone lever for reduction and the installation of a resection guide. The fracture ends were trimmed with a saw, and the surrounding fragmented fragments were removed. A marrow expansion was performed at the proximal end, followed by the placement of the 3D-printed humeral head prosthesis based on the nailing plate guide. Direct visualization confirmed a good match between the 3D-printed prosthesis and the remaining right humeral head. The rotator cuff was sutured onto the 3D-printed prosthesis, and the repair and fixation of the infraspinatus muscle insertion were conducted. Fluoroscopy was utilized throughout the surgery to ensure proper positioning of the prosthesis. Postoperatively, the patient’s condition was favorable, allowing for weight-bearing activities to commence on the second day. The shoulder joint exhibited limited anterior and posterior swinging movements. The surgical incision healed without complications or infection. Once the incision had fully healed, the patient was discharged for home treatment and provided with guidance for rehabilitation training. In the initial phase (0–6 weeks) after surgery, the patient was advised to use a suspension sling (with the option of an abduction pillow) for immobilization. Passive elevation up to 120° was permitted, and pendulum exercises involving forward and backward as well as side-to-side swinging of the affected arm in a bent-over position were recommended. In the subsequent phase (7–12 weeks after surgery), the support device was removed, and resistance training, including external rotation exercises, was gradually introduced. In the third phase (after 12 weeks), comprehensive shoulder joint training was conducted to optimize the patient’s shoulder joint function (Yu et al., 2020).

**FIGURE 3:**
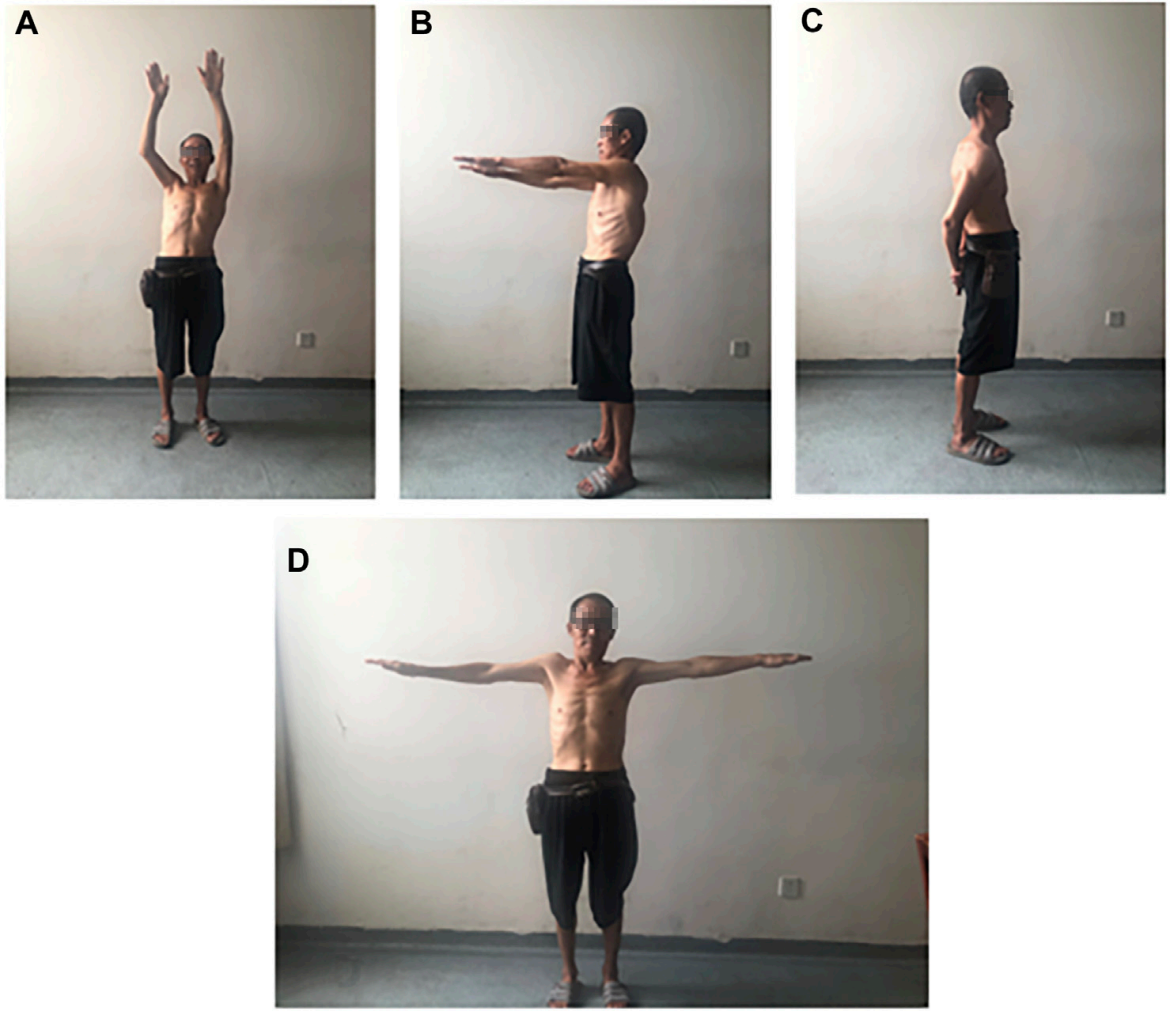
Patient’s range of motion at 1 month postoperatively: **(A, B)** Forward flexion: 90°–150°, **(C)** Extension: 40°, **(D)** Abduction: 90°.

Postoperative follow-up was conducted to evaluate the patient’s recovery. Over a 2-month period at our postoperative follow-up, notable improvements in functional and pain scores were observed between preoperative, 1-week postoperative, and 2-month postoperative assessments. The Visual Analog Scale (VAS) pain score decreased from approximately 5 points 1 week after surgery to approximately 2 points 2 months after surgery. At the 2-month mark, the patient achieved 150° of forward flexion, 90° of abduction, and 40° of extension in the shoulder joint, demonstrating significant improvement compared to preoperative and 1-week postoperative measurements (Figure 3).

**FIGURE 4:**
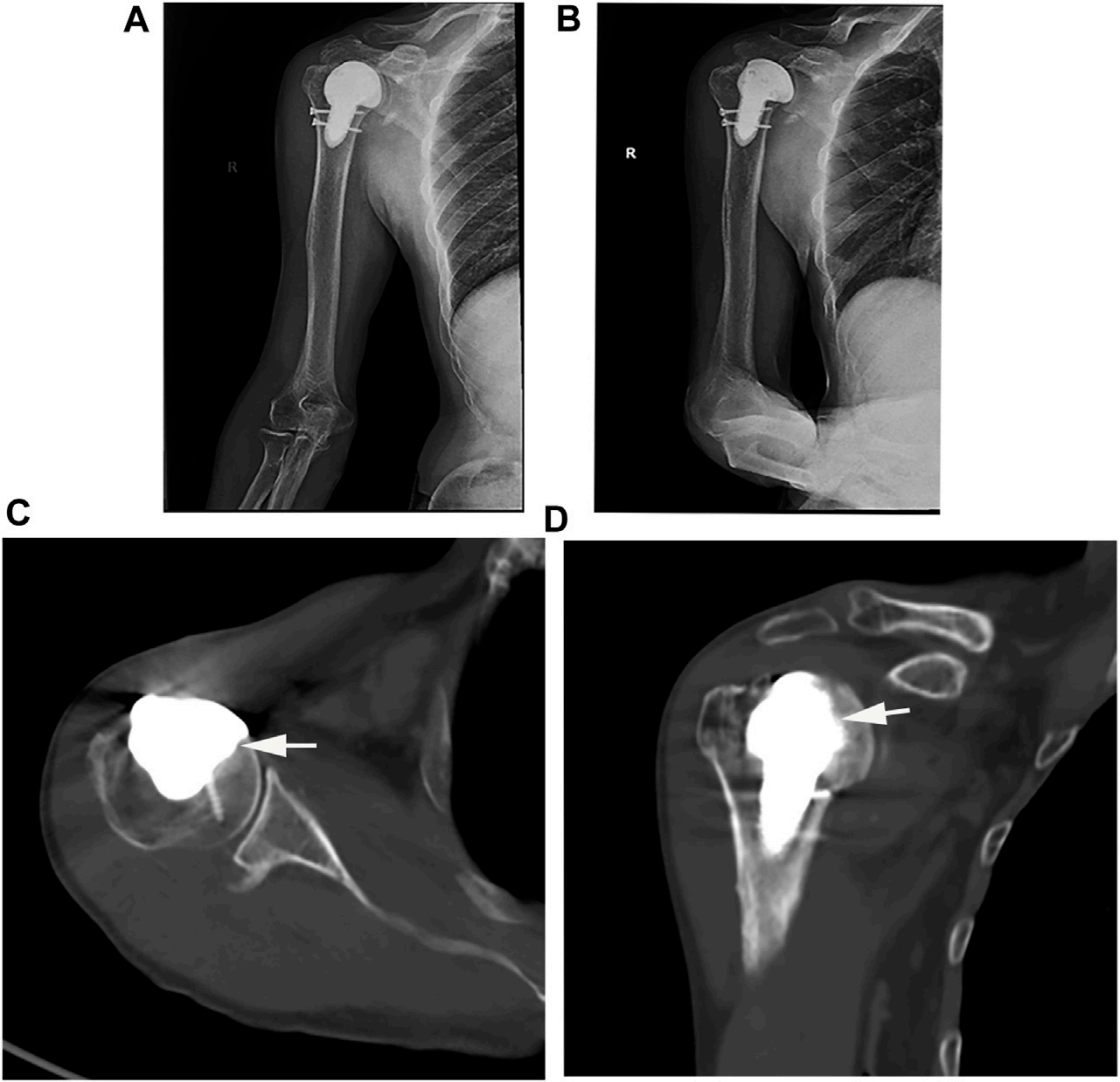
**(A, B)** Postoperative X-ray and **(C, D)** CT examination (White arrow: successful osseointegration).

Postoperative X-rays revealed no evidence of prosthesis loosening (Figures 4A, B). According to the concept of osseointegration proposed by Professor Branemark in the 1960s (Branemark et al., 2001), a permanent bone-to-bone interface, devoid of fibrous tissue intervention, was established when an implant came into contact with actively functioning bone tissue. The occurrence of osseointegration on the prosthesis surface was observed on the 2-month follow-up CT scan (Figures 4C, D).

## Discussion

This case report strictly adheres to The CARE guidelines checklist 2013 edition (Gagnier et al., 2013) and provides a detailed description of a rare case involving an irregular defect of the right humerus caused by an electrical injury, accompanied by posterior dislocation of the right shoulder joint and a reverse Hill-Sachs lesion. In clinical practice, such a condition is relatively uncommon, thus further exploration is warranted regarding its complex etiology and treatment methods for irregular bone defects. In our literature search, we did not find any case reports specifically related to electrical injuries and the use of 3D printing technology for repair and treatment. Therefore, this report represents the first of its kind. However, for the repair of large irregular bone defects, previous studies have demonstrated the potential application of customized repairs using 3D printing technology. However, for the repair of irregular large segmental bone defects, existing research has demonstrated the potential application of customized repair using 3D printing technology. For example, Fiz et al. have suggested that in femoral derotational osteotomy, 3D printing technology is considered a rapid and cost-effective tool for improving surgical outcomes (Fiz et al., 2017).

In this case, the patient suffered multiple electrical wounds on both hands and feet following an electrical shock. The patient also presented with a “locked” posterior dislocation of the right shoulder joint, a reverse Hill-Sachs lesion, as well as a comminuted fracture and a substantial defect of the right humeral head. We believe these injuries were a result of the burns and intense contraction of the muscles around the right shoulder joint. For such conditions, early surgical intervention is crucial to restore the morphological integrity of the humeral head and stabilize the shoulder joint. Studies have shown that conservative treatment can be considered for fresh dislocations with humeral head defects less than 25%. Manual reduction under nerve block anesthesia and the use of external fixation can achieve satisfactory stability of the shoulder joint. However, for patients with humeral head defects greater than 25%, conservative treatment is prone to result in shoulder joint deformity and loss of function, severely impacting the quality of life. Therefore, active surgical intervention should be chosen (Cirino et al., 2022).

Traditional surgical methods for this condition include McLaughlin surgery, modified McLaughlin surgery, shoulder joint replacement, and arthroscopic surgery. Although these methods can effectively restore shoulder joint stability, McLaughlin surgery and modified McLaughlin surgery are only suitable for patients with humeral head defects less than 50% (Yu et al., 2020). They have disadvantages such as long operation time, significant bleeding, extensive trauma, slow recovery, and difficulty in reconstructing bone fragments that fit the defect site. Long-term complications may include bone resorption, traumatic arthritis, and pain. While shoulder joint replacement can address joint mobility issues in the short term, this method may disrupt normal tissue structures and has drawbacks such as long operation time, significant bleeding, and substantial trauma. Additionally, prosthetic implants have a limited lifespan and may require subsequent or multiple shoulder joint replacement surgeries. Arthroscopic repair of soft tissue injuries and restoration of soft tissue balance is suitable for patients with humeral head defects less than 25%, particularly those with defects less than 20% (Martetschlager et al., 2013). This may be due to the fact that in cases with smaller humeral head defects, shoulder joint instability is primarily caused by damage to the joint capsule, labrum, and rotator cuff (Yu et al., 2020). Arthroscopic modified McLaughlin surgery also has some limitations, such as a longer learning curve, uncertain long-term outcomes, and difficulties in reducing locked posterior dislocations under arthroscopy (Du et al., 2023). Therefore, we did not choose these methods.

In this case, the extent of humeral head injury was 40%, with important structures such as the rotator cuff attached to it. The humeral head has a smooth, spherical structure that is difficult to match in shape. Therefore, we chose to use 3D printing technology to design precise implants for the surgery. Unlike traditional internal fixation devices such as plates and screws, 3D-printed implants can seamlessly connect with the remaining portion of the patient’s humeral head, achieving a perfect fit. This not only maximizes the preservation of normal tissue structures, reduces operation time, bleeding, and trauma, but also enables the reconstruction of the proximal anatomy of the humeral bone to facilitate the attachment and repair of a portion of the rotator cuff, thereby improving shoulder joint stability and function. We observed a secure fixation of the implant. Two months post-surgery, we noted the formation of a bony callus at the interface between the implant and the bone, indicating the feasibility of achieving osseointegration with the 3D-printed porous implant. Additionally, 3D-printed porous metal structures can provide excellent bone integration and protection of the subchondral bone and joint surfaces, facilitating bone ingrowth and shaping (Abar et al., 2022).

Regarding the limitation of a 2-month follow-up period, we acknowledge that the follow-up duration was relatively short. However, within the short span of 2 months, the patient’s pain, as measured by the Visual Analog Scale (VAS) score, and shoulder joint range of motion had already shown such significant recovery, further supporting the effectiveness of this technique. Nonetheless, we plan to conduct further follow-up to gain a more comprehensive understanding of the patient’s recovery. Considering the limitations of implants, the presence of implants in the body can endure for several centuries; therefore, the question of whether they will degrade inside the body should not be entertained. However, the surface roughness, morphology, and morphological characteristics of implants can significantly influence cellular behavior, encompassing aspects such as cell adhesion, proliferation, differentiation, and the corrosion behavior of implants. Consequently, most additive manufacturing technologies require post-processing to enhance their surface properties, thereby promoting osseointegration. In addition, challenges such as adhesive removal, sacrificing residual materials, microbial infections, uneven shrinkage, and resolution issues may pose limitations in the manufacturing of these implants (Alipour et al., 2022). As a result, recent research has proposed improved additive manufacturing methods to address these challenges and continues to delve into further studies to better serve patients.

Based on this case, it can be concluded that 3D printing technology offers the following advantages in treating large irregular bone defects of a special nature: 1. Customized individualized implants tailored to the specific factors causing the irregular lesion, enabling personalized and precise treatment to achieve satisfactory expected outcomes; 2. The implant surface possesses a porous and rough trabecular bone structure that better mimics the microstructure of human bones, promoting bone and soft tissue ingrowth; 3. Additive manufacturing techniques allow for customized production of implants that closely match the patient’s needs. Therefore, this technology addresses the shortcomings of traditional shoulder joint surgeries for the treatment of large segmental bone defects and brings benefits to challenging cases.

## Conclusion

The utilization of 3D-printed custom implants for the treatment of irregular humeral head bone defects caused by specific injury mechanisms, particularly in cases of shoulder joint “locked” posterior dislocation and reverse Hill-Sachs injury, represents a promising and advantageous option.

## Data Availability

The original contributions presented in the study are included in the article/Supplementary material, further inquiries can be directed to the corresponding authors.
